# The child and adolescent psychiatry trials network (CAPTN): infrastructure development and lessons learned

**DOI:** 10.1186/1753-2000-3-12

**Published:** 2009-03-25

**Authors:** Mark Shapiro, Susan G Silva, Scott Compton, Allan Chrisman, Joseph DeVeaugh-Geiss, Alfiee Breland-Noble, Douglas Kondo, Jerry Kirchner, John S March

**Affiliations:** 1 Department of Psychiatry and Behavioral Sciences, Duke University Medical Center, Durham, North Carolina, USA; 2 Duke Clinical Research Institute, Duke University Medical Center, Durham, North Carolina, USA

## Abstract

**Background:**

In 2003, the National Institute of Mental Health funded the Child and Adolescent Psychiatry Trials Network (CAPTN) under the Advanced Center for Services and Intervention Research (ACSIR) mechanism. At the time, CAPTN was believed to be both a highly innovative undertaking and a highly speculative one. One reviewer even suggested that CAPTN was "unlikely to succeed, but would be a valuable learning experience for the field."

**Objective:**

To describe valuable lessons learned in building a clinical research network in pediatric psychiatry, including innovations intended to decrease barriers to research participation.

**Methods:**

The CAPTN Team has completed construction of the CAPTN network infrastructure, conducted a large, multi-center psychometric study of a novel adverse event reporting tool, and initiated a large antidepressant safety registry and linked pharmacogenomic study focused on severe adverse events. Specific challenges overcome included establishing structures for network organization and governance; recruiting over 150 active CAPTN participants and 15 child psychiatry training programs; developing and implementing procedures for site contracts, regulatory compliance, indemnification and malpractice coverage, human subjects protection training and IRB approval; and constructing an innovative electronic casa report form (eCRF) running on a web-based electronic data capture system; and, finally, establishing procedures for audit trail oversight requirements put forward by, among others, the Food and Drug Administration (FDA).

**Conclusion:**

Given stable funding for network construction and maintenance, our experience demonstrates that judicious use of web-based technologies for profiling investigators, investigator training, and capturing clinical trials data, when coupled to innovative approaches to network governance, data management and site management, can reduce the costs and burden and improve the feasibility of incorporating clinical research into routine clinical practice. Having successfully achieved its initial aim of constructing a network infrastructure, CAPTN is now a capable platform for large safety registries, pharmacogenetic studies, and randomized practical clinical trials in pediatric psychiatry.

## Review

Clinical research networks are envisioned in the NIH roadmap as a means to reduce the costs associated with launching multi-center studies while increasing patient and physician participation in clinical research, which together should accelerate the pace of medical discovery [1,2]. By clinical trials network we mean a clinical trial coordinating center and a group of clinical trials sites that together are capable of conducting multiple and/or sequential clinical trials and safety registries that where appropriate smooth the progress of biomarker discovery. The Child and Adolescent Psychiatry Trials Network (CAPTN) is among the largest and most sophisticated of seventy-six clinical research networks targeting children and adolescents [3]. The stated mission of CAPTN is to improve the care of children and adolescents with mental illness through innovative clinical research. To accomplish this mission and expand the evidence base in pediatric psychopharmacology, CAPTN seeks to conduct practical clinical trials (PCTs) that evaluate the benefits and harms of widely-used but under-studied medications when conducting such a study that would then serve an important public health need.

In late 2003, we initiated a partnership between the Duke Clinical Research Institute and the American Academy of Child and Adolescent Psychiatry to develop CAPTN as a "proof of concept" PCT network, the first of its kind in psychiatry, adult or pediatric. Funding for CAPTN came from the National Institute of Mental Health (NIMH) through the Advanced Center for Services and Intervention Research (P30) grant mechanism. Since then, CAPTN has conducted one multi-center research study focused on developing and validating a novel drug adverse events detection tool, the Pediatric Adverse Event Rating Scale (PAERS), and is in the process of conducting two linked multi-national studies focused on (1) antidepressant safety and the pharmacogenetics of antidepressant response and (2) a randomized controlled trial of newer versus older treatments for ADHD. The Antidepressant Safety in Kids (ASK: NCT00395213) study is a prospective longitudinal cohort "safety registry" study of predictors of benefits and adverse events in 500 youth with a depressive, anxiety, obsessive-compulsive or eating disorder exposed to an SSRI or SNRI. Running as a substudy within ASK, the Pharmacogenomics of Antidepressant Response in Children and (PARCA: NCT00516932) study is a nested genetic case-control association study evaluating the contribution of selected candidate genes as risk factors for a suicidal event behavioral activation or their association. The Newer Versus Older Treatments for ADHD (NOTA) study is an equipoise-stratified randomized controlled trials comparing for treatments for ADHD: Methylphenidate Transdermal System (Daytrana), Lisdexamfetamine dimesylate (Vyvanse), OROS MPH (Concerta), and Mixed Amphetamine Salts Extended Release (Adderall XR).

At the point we began to seek NIMH funding, CAPTN was believed to be both a highly innovative undertaking and a highly speculative one. One reviewer even suggested that CAPTN was "unlikely to succeed, but would be a valuable learning experience for the field." In actuality, many lessons have been learned in the process of moving CAPTN from concept to functioning PCT network that are of general importance for practical clinical trialists and, in particular, for those seeking to create multi-center clinical trials networks in pediatric and adult psychiatry. In previous reports we documented the need for PCTs in pediatric psychopharmacology [4], described common obstacles to conducting PCTs in psychiatry and proposed a set of solutions [5], and presented a theoretical rationale for CAPTN [3]. In this article, we specifically focus on infrastructure development issues (the "hardware") that enables practical clinical trials (the "software") to run on CAPTN.

## Background

In the October 3^rd^, 2003 issue of Science, the Director of the National Institutes of Health (NIH), Dr. Elias Zerhouni, described a roadmap for clinical research that included three guiding principles believed to be fundamentally important for improving patient care outcomes [1]: (1) develop partnerships among and better integration between organized patient communities, community-based physicians, academic researchers, and the NIH, (2) develop new ways to organize how clinical research information is recorded and make greater use of modern information technologies, and (3) devise new standards for clinical research protocols.

Consistent with the Roadmap initiative, CAPTN is premised on the belief that an expansion of partnerships among stakeholders – academia, pharma, the FDA, the NIH and patient advocacy groups – will facilitate a public-health oriented research agenda that is based on the medical needs of patients and the informational needs of physicians and healthcare policy-makers making patient care decisions [4].

Almost two decades ago, Sir Richard Peto, who coined the term large, simple trial [6], proposed that treatment outcome studies should have sufficient power to identify modest clinically relevant effects, employ randomization to protect against bias, and be simple enough to make participation by patients and providers reasonable [7]. More recently, Sean Tunis at the Center for Medical Services (CMS) described the defining features of an effectiveness trial as comparing clinically important interventions, a diverse population of study participants representative of clinical practice, a heterogeneous practice setting also representative of clinical practice, and a broad range of clinically relevant health outcomes [8]. Building on Peto and Tunis, we recently made the case for practical clinical trials in psychiatry [5]. Consistent with the proposed PCT framework, we formatted the network infrastructure so that PCTs conducted on CAPTN would be characterized by eight defining principles: (1) Questions must be simple, clinically relevant, and of substantial public health importance; (2) PCTs are performed in clinical practice settings; (3) study power is sufficient to identify small-to-moderate effects; (4) treatments should be in clinical equipoise; (5) treatment conditions should be randomly assigned to protect against bias; (6) outcome measures should be simple and clinically relevant; (7) treatments and assessments should enact a best practice standard of care; and (8), subject and investigator burden associated with research should be minimized.

While these characteristics are widely applicable to PCTs in general and, hence, are held in common by practical clinical trialists independent of discipline [9,10], some of the challenges that we faced in developing a PCT network in pediatric psychiatry are specific to the field of pediatric psychiatry itself. Both field-specific and more general challenges provide instructive lessons for those seeking to instantiate the PCT model in that they provide insight into the factors that may inhibit or, conversely, facilitate network creation.

### Network organization and governance

**Figure 1:**
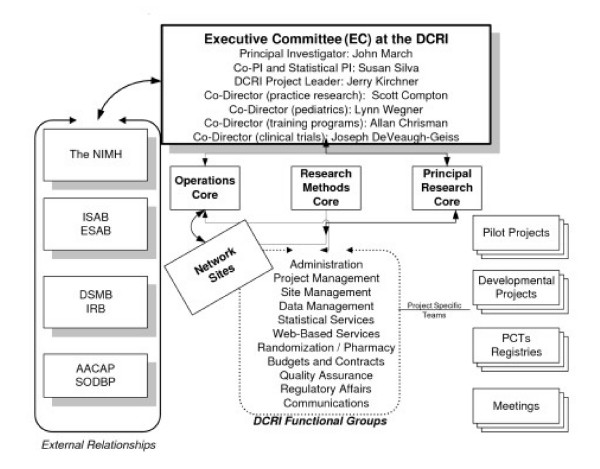
**Child and Adolescent Psychiatry Trials Network (CAPTN)**. Proposed organizational structure for CAPTN during the second five years ofNIMH funding. ISAB = internal scientific advisory board; ESAB = externalscientific advisory board; DSMB = data safety and monitoring board; IRB = institutional review board; AACAP = American Academy of Child and Adolescent Psychiatry; SODBP = Society for Developmental and Behavioral Pediatrics. Pilot projects are small feasibility studies design to gatherpreliminary data to support developmental studies, which are R34 likepreliminary studies intended to construct trial infrastructure and materialsin preparation for R01-like PCTs, safety registries or biomarker/biosignatureprojects.

Figure 1 outlines the CAPTN organizational structure, including trial leadership, governance and coordination, sponsors, advisory boards, partners, network sites, infrastructure capabilities and projects.

#### Executive Committee

Adopting a proper network governance structure is critically important to the overall success of a multicenter clinical trials network. Thus, the first step in creating a collaborative research network, such as CAPTN, was to devise a system for governing the network and enlisting a core group of researchers to take on the managerial challenges. In this regard, CAPTN has benefited from its proximity to many NIH- and foundation-funded research network initiatives based at the Duke Clinical Research Institute. As the oldest and largest academic research organization (ARO) in existence, the DCRI  employs state-of-the-art operational capabilities, including data and site management, biostatistics, and safety surveillance, to facilitate the development, conduct and dissemination of results for NIH and industry funded randomized controlled trials or safety registries. Moreover, the DCRI is a pillar in the Duke Translational Medicine Institute (DTMI: ), which is designed to address T1, T2 and T3 translation blocks identified on the NIH Roadmap.

Thus, we were able to draw upon significant expertise when considering the organizational design of the CAPTN coordinating center. In every organization, leadership must take on the questions of "Who sets the questions or agenda?", "Who has a seat at the table?" and "Where does the final authority lie?" Many network exercises fail because the network leadership reflects either implicit or explicit strategic agreements among powerful site-based principal investigators who as often as not have little or no track record of working together that everyone will "have a piece of the pie." Put differently, many clinical trials networks are (usually covertly) structured to serve the scientific and financial interests of the investigators rather than to meet the mission statement of the network itself. A clear line of authority is necessary to help insure that network members remain stakeholders for the primary mission of the network. Absence of a clear governance structure devoted to the success of the network as a whole, or a failure in process, including means for reconciling disputes, often leads to the formation of subgroups based on self-interests, studies burdened with irrelevant and costly instruments and procedures, sluggish progress on study design and implementation, and poor site and patient enrollment. In developing CAPTN, we explicitly wanted the network to serve the interests of mentally ill children and their doctors. We also wanted clear lines of decision making in which all the major stakeholders in pediatric psychiatry were represented, and we wanted to maximize the public health value of every dollar spent on CAPTN.

From the outset, governance was entrusted to a core group, the CAPTN Executive Committee that is composed of Duke University faculty, the Project Leader from the DCRI, and representatives from the site management, data management and statistics functional groups. With weekly meetings, this group provides a forum for both strategic decision-making and for overseeing the day-to-day operations of the coordinating center ensuring that regular progress is made on all activities essential to the functioning of the network.

#### Advisory Boards

Advising the Executive Committee is a larger group of stakeholder experts: the CAPTN Steering Committee. Prior to the funding of CAPTN, we solicited commitments from key stakeholders in the field of child and adolescent psychiatry. Among these were senior leadership from the AACAP, including representation from both the AACAP Council and the AACAP Workgroup on Research, NIMH Program staff, and a representative from the National Alliance for the Mentally Ill (NAMI). In addition, experts from academia were recruited to offer diverse viewpoints including child psychiatrists, psychopharmacologists, a medical practice researcher, and a pharmacoepidemiologist, all of whom were based outside of Duke University. After funding for CAPTN was received, a representative from the NIMH program office, Dr. Benedetto Vitiello, who consulted to us during the ACSIR application process, was added as an *ex officio *member.

The core members of the Steering Committee were supplemented with rotating at-large appointments from participating network members. More than a dozen physicians responded to our initial request for applicants to join the CAPTN Steering Committee. Selecting applicants for the 2 1/2 year appointments proved to be more challenging than anticipated. Applicants were all highly qualified and diverse in the education, research experience, practice setting, and geographic location, among other factors. Ultimately, the committee selected three applicants who were primarily clinic-based practitioners but each of whom had some prior research exposure. The purpose of this decision was to provide a reality check for the Steering Committee in terms of the additional burdens that we, as researchers, were asking of busy clinicians. While the stated purpose of the Steering Committee was to serve an active if consulting role in managing the network and collaborating on CAPTN trials, the focus on developing and refining the CAPTN infrastructure (a process largely internal to the DCRI) and the fact that the SC members were for the most part too busy to take other than a distant advisory role in CAPTN meant that the SC became in practice more like a traditional external scientific advisory board. Accordingly, going forward, the SC model will be supplanted with a smaller advisory External Scientific Advisory Board.

Our third group of advisors was an internal Scientific Advisory Board. This group was composed of advisors, all from Duke University, in fields ranging from ethics, law, regulation, statistics, medicine, and clinical research. The purpose of this body was to help the CAPTN Executive Committee overcome obstacles or barriers to research that emerged from either internal or external factors. Among the areas that this group advised on were related to structuring contracts, funding for research participation, Federalwide assurances, and ethical oversight for those unaffiliated with an institution with an IRB, bureaucratic inefficiencies within the Duke system, and fitting the mission of the network to the changing regulatory environment. In particular, many of the challenges we faced as a psychiatric network were related to difficulties transporting the mega-trials network model from areas of medicine that take place within facilities with a history of medical research, such as oncology and cardiology, to an area that takes place largely in small, out-patient practice settings.

#### Dispute Resolution

In the rapidly changing landscape of medicine, particularly in the area of drug safety research, the governance structure must accommodate a need for swift dispute resolution, should such disputes arise. Thus, the Principal Investigator was responsible for bringing consensus to the Executive Committee. In cases where consensus could not be reached, the Executive Committee consulted with the Steering Committee. The Scientific Advisory Board was available for consultation about specific challenges or general guidance about factors outside of pediatric psychiatry that nonetheless impacted the network or its research aims. To ensure efficient decision-making, the PI retained final authority over decisions, however with escalation and consultation, the Executive Committee was able to reach consensus over major decisions, the most challenging of which was related to the collection and reporting of Serious Adverse Events (SAEs). This is described below in the section on AE reporting.

### CAPTN Investigators

#### Recruitment

Finding clinicians willing and able to participate in clinical research has been a challenge in many areas of medicine, including child and adolescent psychiatry. Of the 20,000 cardiologists in the United States, fewer than 1000 (.5%) participate in research. Currently, there are approximately 7,000 child and adolescent psychiatrists in the United States and, when we began CAPTN in 2003, only a handful were primarily engaged in clinical research. Thus, the support of the AACAP was critical for our initial and ongoing investigator recruitment strategies. Through a series of surveys, conducted initially at the AACAP annual meetings on paper, and later through the CAPTN web site, we detected a significant level of interest in clinical research among AACAP members: Approximately 300 child and adolescent psychiatrists responded to these early surveys indicating a interest in clinical research within the field, but significant uncertainty as to how one might begin performing clinical research and what additional demands such research would place on clinicians. These surveys were the basis for our initial claim that it would be feasible to recruit and train approximately 200 investigators to join CAPTN and conduct a series of clinical trials. This figure (.3%) is well within the range of research participation by members of the American College of Cardiology, but far smaller than the near 100% participation rate in the Children's Oncology Group, which sees 95% of youth with cancer.

Once CAPTN was funded, we began by contacting the respondents to our initial feasibility surveys. In addition, we reached out through the AACAP to their membership through their web site, email, and at the AACAP Annual Meeting. We asked those interested to complete an interest questionnaire with their contact information and qualifications. During this initial phase of investigator recruitment, we succeeded in identifying 235 child and adolescent psychiatrists who indicated an interest in CAPTN. Including members associated with resident training programs in child and adolescent psychiatry, approximately 150 of these completed the progression from interest to completing a master contract and the CAPTN requirements for human subjects protection. This process took almost three years for reasons outlined below. We also quickly realized that in a group this large, a handful of people in any given month would need to suspend their participation due to circumstances. Ultimately, in a large research network, a certain amount of "churn" is inevitable. In CAPTN, this turns out to be about 10% of the network membership per year either temporarily suspend their participation or permanently do so. Common reasons for this were moves, retirement, and personal reasons, such as divorce or family emergencies. This is an important consideration for those constructing such networks since a steady-state network of 200 investigators must recruit and train about twenty new members per year to sustain itself.

#### Training Programs

Other than at a small number of research-oriented academic medical centers, clinical research has not historically been part of a child and adolescent psychiatrists' residency or fellowship training. Indeed, a survey of clinical trials publications in pediatric psychopharmacology reveals a core group of fewer than 25 principal investigators located mostly at prominent academic centers that are responsible for the majority of recent pediatric psychopharmacology literature. Fortunately, what we noticed from our initial recruitment efforts was that the majority of our members were coming from outside of these major academic medical centers. While this was consistent with our goal of recruiting primarily clinicians, rather than researchers, our long-term network growth strategy was based on reaching out to residency and fellowship training programs. Our intention was to interest trainees in research during their fellowship with the hope that they would choose to continue conducting CAPTN clinical trials after completing their training.

Dr. Allan Chrisman, Child and Adolescent Psychiatry Training Director at Duke, and a member of CAPTN's Executive Committee, developed a training program specific initiative to identify and recruit CAPTN members at facilities with ACGME certified residency training program. Through his efforts at AADPRT and the AACAP Workgroup on Training, we signed up fifteen child and adolescent psychiatry training programs, each of which agreed to engage their trainees in CAPTN research protocols. This outreach coincided with an increased emphasis on research in the accreditation guidelines for psychiatry training programs, and the training we offered was a mechanism for many programs to achieve this target. For research networks, partnership with training programs is a critical strategy for ensuring continued growth in network membership, but more importantly, trainees staffing busy clinics serve as a force multiplier, substantially increasing the capacity of the research network to enroll patients.

#### International Members

Interestingly, while CAPTN was conceived of as a domestic (i.e., United States only) undertaking, our early surveys showed a strong interest among Canadian members of the AACAP. Following discussions with the program office and an application for Fogarty Center clearance, we proceeded with enrolling Canadian child and adolescent psychiatrists and training programs. Although the drugs in use differ somewhat country-to-country, a multi-national expansion of clinical research networks will be essential for achieving the very large sample sizes that have become common in PCTs in, for example, cardiology. Within CAPTN, we are actively working to develop systems for a broader expansion into additional countries over the next few years.

### Regulatory Compliance

One of the main justifications given for clinical research networks is the perceived savings associated with recruiting clinical trial sites once rather than *ad hoc *each time a new study is conceived or funded. To realize these cost savings for a post-marking drug research network, such as CAPTN, we set out to define a minimum set of requirements necessary for investigator participation that would meet applicable regulatory requirements. This activity led to conversations with a wide variety of stakeholders within the Duke community, and ultimately, to a number of investigator and site requirements. The burdens associated with these requirements proved to be a significant barrier to investigator recruitment and participation.

#### Site Contracts

Among these requirements was an executed site contract. Clinical trial site contracting has traditionally been perceived of as both slow and cumbersome, particularly when the contracting offices of major universities are involved. Because of the time and difficulty involved with these negotiations, many in the pharmaceutical and contract research industries eschew academic sites that are not considered to be opinion leaders within the field. Therefore, we had hoped to simplify the process and, in some respects, succeeded, but discovered many barriers to research that were nearly insurmountable within the modern university architecture.

To participate in CAPTN, all site principal investigators must sign a Master Services Agreement. Ultimately, this agreement was pared to seven pages in length but was complicated by several possible combinations of attachments specific to type of facility at which the investigator worked. Under the MSA-model, future studies are all covered under two-page, study-specific contract addenda. Although we hoped for shorter, simpler contracts, we found that most investigators found the proposed language acceptable without modification. However, more lengthy negotiations ensued with other universities. For example, Arizona state law now makes all records of state universities part of the public record. For the participation of state universities in Arizona this necessitated specific language to protect the confidentiality of study records and meant that the study coordinating center had to mark all study-materials sent to the site as "confidential." However, the single largest contractual barrier we encountered was the issue of indemnification and medical malpractice liability insurance.

#### Indemnification

While industry sponsors of clinical research regularly indemnify clinical researchers for activities related to the performance of a clinical study, the Federal government provides no such indemnification for clinical trials. For Duke University's Risk Management office, a large, community-based clinical research network, such as CAPTN, presented a significant potential change in litigation exposure. Ultimately, it was decided that for us to proceed, we had to document the presence of $3 M per incident and $5 M annual aggregate professional liability insurance for all CAPTN investigators. This was coupled with a review by Risk Management related to the solvency of the insurer and history of jury awards within each investigator's locale. While the time involved in collecting and reviewing these documents slowed the contracting process, it turned out that their standards were significantly higher than what is standard within child and adolescent psychiatry, where we found that about 90% of practitioners had coverage of $1 M per incident and $3 M annual aggregate. We were frequently able to obtain waivers on a case-by-case basis for those with less than the required $3 M/$5 M coverage allowing them to participate in those CAPTN studies that were considered non-interventional or minimal risk under 45 CFR 46 Subpart D. This degree of caution is likely to be general for large institutions and those that self-insure since widely distributed geographic exposure and lesser oversight of extramural researchers in a multi-center setting may affect risk exposure and hence, the costs of re-insurance. Ultimately, we were able to proceed with minimal risk research, but this issue reemerged when we began looking to expand CAPTN beyond the United States. These experiences are described below in the section on international research.

#### Oversight: Institutional Review Board

Regardless of the funding source, clinical research must be subject to the oversight of a duly constituted IRB or equivalent body. In the case of CAPTN, the Duke University Health System IRB agreed to serve as the IRB of record for those sites without access to a local IRB. The mechanism to allow this is the Unaffiliated Investigator Agreement (UIA), which was provided to those in solo practice who enrolled in CAPTN. In this arrangement, individual clinicians agree to operate under the purview of the DUHS IRB with the UIA provided as an attachment to the MSA described above. However, it was determined that Duke would not add external institutions to its Multiple Project Assurance (MPA); later replaced by a Federalwide Assurance or (FWA). Thus, group practices and institutions without a local IRB would be required to possess a Federalwide Assurance. The FWA is a binding agreement to conduct clinical research in compliance with the ethical principles outlined in the Belmont Report of 1977 (for those facilities located in the United States) or equivalent (e.g., the Tri-Council Policy Statement in Canada). Thus, for CAPTN, we helped 21 institutions to complete and file an FWA with the Office of Human Research Protection (OHRP). The FWA includes specific terms which the institution must agree to abide by in order to receive the assurance. In addition to identification of a Human Protection Administrator, the facility must adopt written policies and procedures to ensure compliance with ethical and regulatory requirements. While CAPTN has helped facilities develop these procedures, some potential CAPTN participants balked when confronted with the time commitment necessary to complete the training and adopt the formal procedures required.

#### Regulatory Documentation

In addition to the contractual demands of research, another significant burden is the collection and maintenance of essential regulatory documents. For studies conducted under an IND pursuant to 21 CFR 312, this documentation includes a form FDA 1572, investigator resumes or curricula vitae, and medical licensure for every site participating in the research. Finally, in psychiatry, and in particular, in child and adolescent psychiatry, use of medications scheduled under the Controlled Substances Act is commonplace (for treatment of Attention Deficit Hyperactivity Disorder). Since it was highly probable that we would conduct studies in this area, we opted to require network members to have prescribing authority under the CSA. This is commonly documented by the form DEA 223.

To date, CAPTN studies have not required an IND, although this might be expected in some cases, since many, if not most, medications used in this area are approved for use in adults, but not in children. Therefore, we felt that the collection and maintenance of regulatory documents should be sufficient to meet the requirements of an IND study, following the stipulation of 21 CFR 312.53 and ICH 4.1.1 which require that sponsors of research document that clinical investigators are qualified to conduct studies of the investigational product. For CAPTN, this has been defined to mean those physicians who are licensed in good standing to practice medicine and have completed specialty training in psychiatry and child and adolescent psychiatry. We documented this with information from the American Board of Medical Specialties (ABMS) and AACAP, whose databases allow us to verify that our investigators have completed the required residency training and board certification. However, as an NIH-funded enterprise, CAPTN is also subject to the requirements of 45 CFR 46.

For federally-funded studies, an NIH format BioSketch takes the place of curriculum vitae. In addition, human subjects' protection training and a Federalwide Assurance (for studies that are more than Minimal Risk under 45 CFR 46 Subpart D) are also required. For community sites, the human subjects' protection in research training requirement is typically met with the on-line module entitled "Human Participant Protections Education for Research Teams." This module is available from the National Cancer Institute through their web site at . It is important to note that many Institutional Review Boards (IRBs) require their own training modules which we allowed as substitutes for this training by those operating under a local IRB, if the local IRB was registered with OHRP and the institution had an active FWA. This required training takes approximately an hour to complete on-line and provides a certificate documenting completion of the training. For a small fee Continuing Medical Education (CME) credit is available.

Clinical researchers may be audited by any of several agencies that are empowered to restrict their ability to participate in clinical research. Among these are the OHRP, NIH Office of Civil Rights, and the FDA's BioResearch Monitoring Program. We require that all members be unrestricted by any of these regulatory agencies with respect to their ability to participate in human subjects' research.

#### Managing Investigator Burden

As these requirements became defined and we received feedback from network members, it became clear to us that the requirements were excessively burdensome. Surveys of those members who completed all requirements indicated that the time required by investigators to provide documentation that they had fulfilled these requirements was estimated to be ten to fifteen hours. Note that this does not include any training for specific protocols. Ultimately this proved to be a drag on investigator recruitment and was complicated by the fact that CAPTN wasn't budgeted to cover the time costs of these requirements.

For experienced researchers working in facilities with support infrastructure, such as a clinical research coordinator, ten hours may not seem onerous, but for CAPTN members, 80% of whom are in private practice, additional paperwork of this magnitude was seen as an overwhelming barrier to participation. Consider that, in private practice, and particularly, in solo practices, every hour spent on these activities means unseen patients and an opportunity cost to the practice. Child and adolescent psychiatry is a field where the wait to see a physician is six months in many areas, meaning the time required to meet these requirements occurred at a loss to patients, too. Recognizing that the burdens were more time-consuming than we anticipated, we discussed the situation with our Steering Committee, Scientific Advisory Board, and the program office at the NIMH, and made a decision to offer reimbursement for the costs of research above the costs of clinical care. While we recognize that the costs of this time differ based on overhead costs in different regions and practice settings, we settled on reimbursement at $150 per hour. This number was arrived at through both surveys and a consideration of our ability to pay, based on re-budgeting of the network coordinating center's funds away from other activities.

As the burden of regulatory and other requirements became clear to us, we recognized that innovative strategies to lessen these burdens were imperative. We achieved substantial cost savings by automating the collection of regulatory information and moving this burden from investigators to the study coordinating center. Ultimately, the system we developed led to an enormous cost savings and represents a significant advancement in recruitment of clinical investigators. This system is described below.

In recent years, an increasing amount of information has become available in electronic format. Although clinical research and medicine in general has lagged other business in terms of adoption of information technologies, many organizations are actively moving towards electronic records. For CAPTN, we linked our web-based enrollment survey system to a live database that integrated physician licensure information from the Federation of State Medical Boards portal. Using custom scripts for each state's web site we were able to quickly automate collection of licensure information for about 85% of the U.S. population. We combined this with imports of child and adolescent psychiatry training, demographic, and contract information from the AACAP database, board certification information from an automated script for the ABMS web site, DEA licensure information from the DEA registrant database available from the National Technical Information Service (NTIS), and FWA information from the OHRP. This system enabled us to quickly document investigator qualifications from reliable and valid information sources.

When an investigator signs up for CAPTN through our web site, we are able to match up the information provided on our short enrollment survey with this composite database to verify that their credentials are current and sufficient for membership in CAPTN. The information from this survey is then merged into the NIH format BioSketch and an email with a link to the required HSP training.

While this system, an aggregate of more than sixty live and static databases, represents a significant innovation in terms of documenting investigator qualifications under 21 CFR 312 and ICH 4.1.1, we feel that it may be a starting point for easing the burdens of research across specialties. Specifically, we envision a similar system operated centrally by the NIH or FDA in a manner analogous to FWA and IRB registries currently maintained by OHRP. A central system, linking qualification databases such as medical licensure, residency training and board certification, and DEA registrant information together with the HSP training records database at the NCI and institutional information from OHRP (IRB, FWA) could vastly simplify the process of documenting investigator qualifications for research, and provide a means for centrally tracking research participation by oversight bodies within DHHS. A convenient platform for such a project already exists, in part, through the . The benefits of joining these systems into a large clinical research information architecture are that investigators would be relieved of providing duplicate regulatory documents for each trial that they participate in to sponsors who are required to collect and process them. This reduced the overall burden of research and facilitates the work of oversight agencies and IRBs by allowing real-time tracking of qualifications and HSP training along with study participation.

### Professionalism: Training Clinicians to be Clinical Researchers

A central myth in the field is the belief that being an expert clinician entitles one to do treatment research. Nothing could be further from the truth. While it is very helpful to have good clinical skills, the research skills necessary to be a Good Clinical Practice (GCP) compliant clinical trialist or to effectively run a GCP compliant clinical trial site are separate from the basic skills required for clinical practice.

Accordingly, CAPTN, which by intent recruited investigators without research experience, required us to put in place a variety of vehicles for terrific clinicians to become terrific research sites. In addition to the NCI's training or training offered by a local IRB, we made research ethics training created by Duke University's Trent Center for Bioethics, Humanities, and the History of Medicine freely available to all CAPTN investigators. These research ethics training modules cover a wide variety of the ethical and scientific aspects of performing human subjects' research. The modules also provide free continuing medical education (CME) credit. We felt that this was one potential benefit of participating to physicians participating in CAPTN.

In addition to HSP training, we partnered with the Communications Department at the Duke Clinical Research Institute to offer a ten module series of on-line training in clinical research through the CAPTN web site. This training is based on the clinical research textbook, Lessons from a Horse Named Jim. This textbook was provided free of charge to all CAPTN investigators after execution of the network MSA. Our aim was to provide both the book as a reference and a tool for self-directed training in clinical research. In the first two years that the on-line training modules were available, fewer than a dozen network members completed any of the training modules. This may have been due, in part, to the fact that initially CEU, but not CME was available for completing the modules.

In fact, the cost and requirements for obtaining accreditation for these modules to provide CME was a barrier for the coordinating center. We found that working through the AACAP, which is an authorized provider of CME, simplifies the offer of CME credit for training modules created by CAPTN. Recently, we have completed modules on Understanding in Incorporating Evidence-Based Medicine into Clinical Practice and Understanding Issues of Diversity in Clinical Research and Patient Recruitment. Additional modules are in development.

Cumulatively, CAPTN members were required to complete about two hours of training prior to participating in our first clinical research study. This training was delivered via the web-based modules, teleconferences, and web casts. Our experience in the first Network Initiation study suggests that this training alone is not sufficient for new clinical researchers.

### Study Monitoring

One of the main costs, and thus barriers to, expanded clinical research within the context of medical practice is the cost of monitoring compliance at clinical sites. ICH 5.18.3 requires monitoring of clinical trial sites, but doesn't define a minimal level of monitoring of research studies. Since IRBs are required to review studies at least annually, this has become the minimum standard for monitoring of clinical research sites. Historically, clinical research monitors (CRAs) have traveled to sites to verify source documents, conduct training or retraining, and assess compliance with ethical and regulatory guidelines. For our first Network Initiation study, we required internet-based research ethics training and conducted a series of web cast protocol training sessions that lasted about one hour each. Coordinating Center personnel contacted sites several times per month using phone, email, and/or fax to answer questions, collect enrollment information, and discuss the conduct of the study. Over the next year, we physically monitored all sixty-five sites that participated focusing on ethical and regulatory compliance. The findings of these activities showed a large number of issues with the performance and documentation of informed consent at approximately half of PAERS sites.

Since most investigators were research naïve prior to this study, we discovered *post hoc *that an hour each of ethics and protocol training was insufficient for most clinicians with respect to the execution of the informed consent process. After working with sites to report these findings to their IRB, we determined that the available training did a good job of educating clinicians about the regulatory and ethical aspects of doing human subjects research, but did not give them a framework for implementing procedures to comply with these guidelines. In response, we created SOPs to help sites enact procedures that would allow them to meet the requirements. In addition, we have embarked on creating a professionally-produced, web-based *interactive *training that is scenario-based and makes extensive use of video. This educational module is currently in production and will be completed and placed in the public section of the CAPTN website in Quarter 1 of 2010.

Following this study, we surveyed participating investigators and discussed the findings with our Steering Committee. The at-large members were particularly helpful explaining to the Steering Committee the specific burdens created for doctors when they had to add additional time to their patient care encounters (for example, for conducting informed consent discussions) or increase their overall paperwork burden. Indeed, three factors here are particularly relevant for those attempting to increase participation in medical research by physicians. First, pressure from payers has reduced the amount of time child and adolescent psychiatrists (and other specialists) are able to spend with patients. Aggravating this is the severe shortage of child and adolescent psychiatrists in North America. Our surveys of participants and potential participants indicated that their initial encounters with treatment-seeking children and families lasted a median of one hour. For medication management visits, the median time spent with a patient was twenty-five minutes. CAPTN reimburses for the cost of research above the cost of routine clinical care. After conducting our first study, we found that it took an average of 45 minutes time to conduct the informed consent process with the family, approximately thirty minutes to complete the research portion of the clinical interview, and twenty-to-thirty minutes to complete the case report forms required for our study. While this is a tiny time allocation relative to efficacy/effectiveness trials like the Treatment for Adolescents with Depression (TADS) study[11,12], it is not inconsequential for busy physicians. Hence, for participation in research to be financially neutral for physicians, studies must be budgeted accordingly, and the budget for site-based payments must be large enough to absorb the sample sizes required in practical clinical trials.

### The Data Safety and Monitoring Board

In addition to field monitoring for regulatory and ethical compliance, a Data and Safety Monitoring Board (DSMB) was established as an independent body to periodically evaluate the progress of CAPTN studies. This includes a review of safety data for signals that might warrant modification, suspension, or discontinuation of a particular study. In developing a charter for the Board, we made extensive use of the NIH Policy on Data and Safety Monitoring, FDA's draft guidance on the establishment and operations of Clinical Trial Data Monitoring Committees, and the DAMOCLES study group's guidelines. In addition, members of our executive committee drew on their own experiences chairing or serving on numerous DSMBs/DMCs for NIH and industry.

When recruiting members of this board, the issue of liability insurance reemerged. Although lawsuits against DSMB members are rare, and have not, to our knowledge, been successful, this remains a possibility and a barrier to recruiting qualified candidates. This is specifically since it is recommended that a majority of members be independent of the sponsoring institution (Duke, in our case). Those serving on the board with appointments at Duke would generally be covered under Duke's insurance but Duke generally does not indemnify independent contractors, such as DSMB members, meaning that the external members should either come from an institution that would cover them or they must purchase their own coverage. As the importance of DSMBs increases, we anticipate more difficulty in recruiting qualified candidates. To alleviate this, liability insurance or indemnification should be made available to those agreeing to serve on these boards.

We required that members of our DSMB submit annual Conflict of Interest and Financial Disclosure certifications modeled after the policy for such disclosures at Duke. The requirements for this disclosure stem from 21 CFR 54 and, for NIH-funded projects, such as CAPTN, 42 CFR 50. The purpose of these disclosures is to ensure that those participating in CAPTN research are fully objective.

Based on Duke's policy and the guidance available from the NIH, FDA, and International Council of Medical Journal Editors, we crafted a financial disclosure and conflict of interest (COI) policy for the network that is currently applicable to CAPTN publications and CAPTN faculty. Respecting the balance between the ethical and regulatory need for full disclosure of competing interests and the additional burden full disclosure might place on CAPTN investigators participating in CAPTN research projects, we are currently considering extending conflict of interest reporting to all CAPTN investigators using the CAPTN practice profiling tool, the Practice Research Survey.

### Study Architecture

To realize the efficiencies of a research network, we sought to develop a standard platform on which to capture clinical trial information. This core Case Report Form battery consisted of basic patient, practice, and diagnostic information for screening and enrollment, along with a standard treatment-level form that included validated and reliable measures of safety, tolerability, and efficacy.

#### Diagnostic and Outcomes Assessment

After extensive research, we chose the youth- and parent-reported DISC Predictive Scale, version four (DPS-4) as our diagnostic instrument. The DPS-4 contains a series of questions drawn from the Diagnostic Interview Scale for Children that can be answered with a simple yes or no. The information provided can quickly be scanned by a clinician to validate their clinical interview findings. Overall, the use of scales should be part of the best practice standard-of-care, but such scales should not add extensive time to the patient care encounter, or they would become unrealistic in clinical practice. To improve usability and to provide comparability to European PCTs, we considering a switch from the DPS to the Health of the Nation Outcome Scale for Children and Adolescents (HoNOSCA), which comes in child, parent and clinician formats, is shorter and simpler to administer and score than the DPS, and is a proven clinical trial endpoint [13-17]

For evaluation of effectiveness of interventions, we chose the Clinical Global Impressions, Severity and Improvement scores (CGI-I, CGI-S) and the Child Global Assessment Scale (CGAS). We ask clinicians to rate the severity of primary illness, that for which study treatment was initiated, overall mental illness, and global functioning. Where appropriate, we also ask the investigator to provide Clinical Global Impressions-Tolerability (adverse event burden) and Acceptability (formulation acceptability) scores, which when combined with a CGI-I score allow the investigator to provide a composite CGI-Effectiveness score based on the balance of benefits, tolerability and acceptability. Together, these simple measures provide a reliable snapshot of the subject's diagnostic status and functioning over time.

#### Adverse Event Monitoring

Adverse events (AEs) following treatment with psychotropic medication are a common but understudied cause of iatrogenic morbidity. Drug-induced, adverse events (AEs), including suicidal events, are more common in adolescents than in adults, and more common in children than in adolescents. However, despite the availability of valid and reliable PCT friendly diagnostic and endpoint assessments, we found no suitable measure available for adverse event elicitation. This is consistent with recent critical reviews conducted by the Research Units on Pediatric Psychopharmacology (RUPP) indicate that little is known about the incidence and prevalence of AEs, especially less common events and, until recently, the field has not had a standardized procedure for prospectively ascertaining common, rare or unique treatment-specific AEs [18-20].

Consistent with GCP standards and the goals of CAPTN itself, safety monitoring is a primary aim of most if not all CAPTN trials. To accomplish safety monitoring with the framework of the CAPTN eCRF, we required (1) an AE monitoring tool to identify and record common AEs and (2) a mechanism for recording severe harms that would be informative from a data perspective and would satisfy DSMB/FDA requirements for serious adverse event reporting. To this end, using CAPTN as the platform, we developed and psychometrically validated a new AE rating scale, the Pediatric Adverse Events Scale (PAERS), which is designed to allow rigorous prospective identification of AEs in a PCT framework [21,22]. Fully integrated with the CAPTN eCRF, the PAERS addresses the following issues: (1) simplicity and ease of use; (2) clinical applicability to a wide variety of drug exposures; (3) frequency, severity, and subjective importance of an AE; and (4) empirically-derived, psychometrically validated item content. The PAERS, which can be administered at every visit or at selected visits, is a 45 item AE inventory estimated to take 10–15 minutes to complete. Forty-three items contain empirically-derived and validated item content and 2 items are open. The PAERS includes child and parent forms intended to set the prior probabilities for the clinician to formally ascertain AEs the baseline and all post baseline visits. Absent, a child or parent form, the PAERS includes a clinician interview option. All three versions use a Likert-style response format in which the informant indicates how much each item (sign or symptom) was bothersome or a problem. Having reviewed the items with the patient and parent, the clinician records all AEs and summary information on the PAERS clinician form including specifying presence in the past week, resolution, attribution, severity, and impact on function. Both formats – child/parent and clinician interview – are acceptable to doctors and patients and, hence, are frequently used by CAPTN investigators in clinical practice.

While the PAERS provides an acceptable level of detail for typical adverse events, it does not adequately assess serious or harm-related events. Thus, having reviewed the PAERS, the study investigator also completes a separate screen for parasuicidal behavior, suicidal ideation and behavior, harm to others, and medical and psychiatric serious adverse events. The eCRF also includes a section on behavioral toxicity, including but not limited to behavioral activation, agitation, akathisia, disinhibition, inhabitation, emergence or worsening of ADHD, and the serotonin syndrome. Any or all of these or any severe adverse event may then trigger the CAPTN eCRF SAE/Harm Form. If the event is serious non-psychiatric (e.g. hospitalization for a femur fracture), psychiatric (e.g. mania or severe panic), or harm-related (suicidal event or harm to others) so some combination of these, the clinician will record systematic information in checklist form, including: narrative description, seriousness assessment (FDA SAE checklist), relationship to study medication, event status, event characteristics, relationship between multiple events, harm to self/others type and detailed characterization, contributing factors, and event resolution. To avoid the necessity of event adjudication, the CAPTN eCRF specifically provides for event coding under the FDA's rubric for seriousness and the Columbia Suicide coding scale[23,24]. Events are held open and tracked until resolved. Branching logic allows for relatedness across compound SAEs. At the next visit, an event resolution workflow is automatically triggered until the event is resolved or the patient exits the study.

#### The CAPTN eCRF

After gathering feedback about the CRF and assessment measures, and extensive analysis of the study data, we revised and finalized our core CRF battery. This battery was then built into an electronic format using Clinipace's TEMPO electronic data capture (EDC) system. This system allowed us to make extensive use of logic checks and branching to minimize the number of questions an investigator would need to answer about a study patient, based upon their answers to pervious questions. The validation allowed us to enact complex logic checks on screen, eliminating the need for *post hoc *queries which are not feasible in a PCT setting. This system is currently being used in the CAPTN Antidepressant Safety in Kids (ASK) trial as well as in a pharmacogenomic sub-study looking at genetic predictors of suicidality and behavioral activation, among other endpoints, and will be portable with minor modifications for all future CAPTN practical clinical trials. A standard platform, shared across the network allows a significant cost savings across studies.

## Conclusion

It is widely acknowledged that clinical trials in psychiatry frequently fail to maximize clinical utility for practicing clinicians, or stated differently, available evidence isn't perceived by clinicians (and other decision makers) as sufficiently relevant to clinical practice, thereby diluting its impact [4]. To maximize clinical relevance and acceptability, researchers in other areas of medicine – such as cancer and cardiology – have turned to practical clinical trials, which are always simpler and usually larger than conventional efficacy clinical trials [8]. We recently made the case for transporting the practical clinical trials model to psychiatry, defining requirements for stable funding for network construction and maintenance plus methodological innovation in assessment, treatment, data management, site management, and data analytic procedures. [5].

During its first five years, CAPTN successfully addressed the aims of the NIH roadmap. Specifically, we have made extensive use of information technologies to developed innovative solutions to reduce the burden and cost of clinical research. In addition, were have responded to the charge of setting the research agenda with input from a wider array of stakeholders by partnering with the AACAP and enlisting representatives from advocacy organizations. Together, these have allowed us to move the field forward and prove the feasibility of PCTs as part of an overall move towards increasing the use of EBM in the field of child and adolescent psychiatry. Over the next two years, CAPTN will complete the largest safety registry ever in its field – a 500+ subject patient safety study of antidepressants that will include specific measures for safety monitoring and evaluation of behavioral activation and its putative link to treatment-emergent suicidality. This will be coupled with a pharmacogenomic study to assess genetic markers for antidepressant benefits and harms. In addition, CAPTN is in the process of conducting an equipoise stratified randomized comparison of newer versus older medications in ADHD. The information gained will meaningfully improve the ability of both clinical and policy decision-makers to make decisions in the best interests of youth with mental illness.

In response to PAR-08-088 for Advanced Centers for Interventions and/or Services Research (ACISR), the CAPTN team is in the process of applying for a second five years of NIMH funding. If successful, CAPTN will complete ongoing studies; expand to 250 active participants including an extension into Developmental and Behavioral Pediatrics; restandardize and disseminate the PAERS; initiate a pioneering programs of research in diversity, office-based parent management training in ADHD, and adaptive treatment strategies in pediatric bipolar disorder; add collaborative NIMH- and industry-funded clinical trials and in so doing open the Network to outside researchers wishing to use CAPTN to conduct PCTs; conduct jointly with the NIMH five field-leading workshops on topics relevant to practical clinical trials in pediatric psychiatry; and expand career development initiatives begun during the first five years of funding. Accordingly, CAPTN promises to enhance the public health relevant evidence base in pediatric psychopharmacology and to further develop the capacity to conduct practical clinical trials in mentally ill children and adolescents.

## Competing interests

Mark Shapiro has no competing interests to disclose.

Susan Silva is a member of a DSMB overseeing research conducted by the NIMH. She has no other competing interests to disclose.

Scott Compton is a scientific advisor for the TSA Clinical Trials Consortium. He is a member of a DSMB for NIH Funded Trials.

Allan Chrisman is a consultant or scientific advisor for Eli Lilly, Addrenex and the NIMH.

Joseph DeVeaugh-Geiss is a scientific consultant for Voyager Pharmaceuticals, Schwarz Biopharma, Febre-Kramer Pharmaceuticals, Pozen Pharmaceuticals, GlaxoSmithKline, Jazz Pharmaceuticals, NuPathe, CeNeRx BioPharma and JDS Pharmaceuticals. He has equity holdings (owns stock) in GlaxoSmithKline and Pozen Pharmaceuticals, and Voyager Pharmaceuticals. He receives consulting fees from the venture capital companies of Scale Venture Partners and Sofinnova Ventures.

Alfiee Breland-Noble receives > $25,000 under separate independent grants from NIMH funded clinical trials.

Douglas G. Kondo receives minor research support from Repligen, Inc.

Jerry Kirchner has no competing interests to disclose.

John March is a consultant or scientific advisor to Eli Lilly, Pfizer, Wyeth and GlaxoSmithKline; an equity holder in MedAvante; the author of the Multidimensional Anxiety Scale for Children (MASC); a member of a DSMB overseeing research conducted by Astra-Zeneca and Johnson & Johnson; conducts sponsored research for Pfizer: and under separate independent grants receives study drug from Eli Lilly and Pfizer for two NIMH-funded clinical trials. Dr. March does not participate in Speaker's bureaus or other promotional activities.

## Authors' contributions

With JM as the senior investigator and author, all authors contributed to the design and implementation of CAPTN and to the conceptualization and writing of this manuscript. All authors read and approved the final manuscript.

## Corresponding Author

John S. March, MD, MPH, Director, Division of Neurosciences Medicine, Duke Clinical Research Institute, Duke University Medical Center, Durham, NC 27705, Email: john.march@duke.edu.
